# 26^th^ Panhellenic Rheumatology Congress, 6–9 December 2018, Athens, Greece

**DOI:** 10.31138/mjr.29.4.190

**Published:** 2018-12-18

**Authors:** Spyros N. Nikas

**Affiliations:** Private Practice Rheumatologist, Ioannina, Greece

**Keywords:** Panhellenic Rheumatology Congress, Highlights

The 26^th^ Panhellenic Rheumatology Congress, which is organised by ERE-EPERE (Greek Rheumatology Association and Health Professionals Association of Greek Rheumatologists, http://ere.gr/), took place on 6–9^th^ December 2018 in Athens, Greece. This four-day biennial meeting represents the most important Rheumatology event in Greece. This year, it was attended by 372 health professionals from academic, public, and private medicine backgrounds, 64 medical students, and strategic development directorates from at least 21 pharmaceutically-sponsored companies. The attendees had an opportunity to update their knowledge concerning the majority of autoimmune, inflammatory or non-inflammatory rheumatic diseases.

The congress was honoured by the presence of the President of EULAR, Johannes Bijlsma, EULAR’s past president Maxime Dougados, and other invited honourable foreign guests - Monika Østensen, José António Pereira da Silva, Peter Taylor and Marios Kouloumas (EULAR past vice president). More than 67 scientific lectures were delivered. There were also workshops and meetings of special interest, such as practical sessions on joint and soft tissue injections, musculoskeletal ultrasound hands-on trainings, “meet the expert” neurology and lupus nephritis sessions, vasculitis and imaging cases, internal medicine issues (e.g., liver involvement, infections in rheumatic patients, neoplasms in rheumatic diseases), soft tissue rheumatism, lumbar spine problems and arthroplasties, sessions on how to organise Private Medicine Rheumatological Offices, and the 3^rd^ forum of Rheumatic Diseases Stakeholders.

In his opening lecture (*“Where we are and where we are going”*), Dr Bijlsma talked about current state and future perspectives of rheumatoid arthritis (RA) studies. He presented findings about prevention and genetic susceptibility, epigenetic modifications, and environmental factors such as smoking and infections. Nowadays, IFN and B (B27) cell signature and early MRI imaging (bone marrow oedema) in patients with early arthritis and arthralgia are viewed as promising predictive tools to identify those at risk of RA: there are still unmet needs in issues such as the prediction of who “will get RA”, or how effectively we are able to treat patients that might “get RA”.

Dr Petros Sfikakis reflected on the importance of real-world evidence (registries, case reports, case series, pharmacovigilance). He mentioned that 2 out of 10 patients with prescribed biologic therapies are eligible for RCT. In the same session, data from PSABIO study (*Ustekinumab and Tumour Necrosis Factor Alpha Inhibitor Therapies in Patients with Psoriatic Arthritis in Standard Health-Care Practice; A Prospective, Observational Cohor*t), with comparable findings with TNFi or ustekinumab, including data from Greek patients, were presented.

Dr Østensen from Sorlandet Hospital, Kristiansand, Norway highlighted potential risks from uncontrolled maternal disease during and after pregnancy for mother and child. Different treatment needs across rheumatic diseases and pregnancy outcomes in inflammatory arthritis, compared to healthy population, were also discussed. Compelling evidence suggests that there is no difference in pregnancy outcomes when TNFi-exposed pregnancies were compared to disease-matched controls.

An outline of “modern face of an old disease” was presented by Dr Xenofon Baraliakos (Germany), who highlighted how important the “treat to target” (T2T) approach is in patients with axial spondyloarthritis, implying clinical remission. The issues of improved screening methods, diagnostic vs. calcification criteria, and prediction of therapeutic response were discussed by the expert.

The concept of T2T in RA was highlighted by Dr Dougados. He clarified issues related to specific disease targets (DAS28, corticosteroids), outcome measures (concomitant fibromyalgia), treatment impact on imaging progression, (increased osteoblast reaction in areas of fat metaplasia) individualized approach, or even combination therapies (bDMARDs plus physiotherapy or NSAIDs), and the latest evidence (5 out of 6 six trials showed superiority of a T2T approach versus routine care, which leads, in addition to better pain outcomes, to less comorbidities and cardiovascular risk). Data from a Greek survey were presented by Dr George Kitas, who demonstrated that Greek rheumatologists support the T2T approach; the majority of the specialists (37%) believe that the achievement of a specific target is the most important issue in the treatment of RA and spondyloarthritis. The T2T approach in axial, peripheral spondyloarthritis and psoriatic arthritis (PsA) was also highlighted by Dr Theodoros Dimitroulas. He discussed treatment goals in PsA (DAPSA score <4 for remission as a better validated index compared with functional status and structural progression; and MDA [Minimal Disease Activity] with 5 out of 7 criteria met). He also discussed novel tools such as PsAID12 (EULAR Psoriatic Arthritis Impact of Disease).

New data about several autoimmune rheumatic diseases were presented by Dr George Bertsias. He pointed out that the new EULAR/ACR classification Criteria (presented at ACR 2018, basic inclusion criterion ANA [Hep 2 IIF] ≥1:80, score ≥10) are not diagnostic criteria. The effects of hydroxychloroquine in lupus patients were discussed in relation to disease pathogenesis, skin, joints, disease relapses, glucose and lipids profiles, platelet activation, thrombosis, and mortality. Data about therapies with belimumab, mycophenolate mofetil plus voclosporin, rivaroxaban in antiphospholipid syndrome were touched upon as well. Exciting data were presented on behalf of the Greek Registry of Birth from Patients with Systemic Lupus Erythematosus. Most of these births have successful outcomes, with slight trends in prematurity or low birthweight.

Dr Alexios Iliopoulos raised the issue of “Osteoporosis Crisis”, showing the details about trends in osteoporosis screening and treatment (significant reduction of bisphosphonates prescription from 2008 onwards in USA). He also analysed data on cardiovascular safety of calcium intake, and referred to the adequate existing evidence on benefits of vitamin D, calcium, and combined supplementation for preventing fractures. The recent update of guidelines from the American College of Physicians on treatment of Low Bone Density or Osteoporosis were presented (pharmacologic therapy for 5 years, no bone density monitoring during this 5-year pharmacologic treatment), as well as data about “drug-holiday” (special warnings for teriparatide or denosumab) or ongoing protective bisphosphonates’ role after treatment discontinuation (alendronate for 2–3 years, risedronate for 1–2 years). Finally, the efficacy and safety of modern therapeutic agents such as romosozumab (humanized monoclonal antibody that targets sclerostin) were discussed.

Of special attention were the presentations of novel treatment approaches, including ixekizumab (IL-17A specific monoclonal antibody) in biologic-naïve patients with active psoriatic arthritis (phase III trial SPIRIT-P1, 80mg/4w: ACR20 58%). Clinical effectiveness was independent of methotrexate co-administration, with injection site reactions being the most frequent adverse events. SPIRIT-P2 phase III trial was analysed, providing data of the efficacy (24wACR20 53%) and safety of ixekizumab in patients with active psoriatic arthritis with previous inadequate response to TNFi.

Recent findings of MEASURE 1 study were presented by Dr Dimitrios Daoussis. He pointed that secukinumab therapy is associated with low radiographic progression and sustained efficacy over 4 years in patients with anky-losing spondylitis (ACR18). Similar results were reported based on another 4-year extension phase III study of this agent in patients with PsA (FUTURE 2), with encouraging findings of resolution of dactylitis (88%) and enthesitis (71%) through week 208. The presence of these new therapeutic alternatives in patients with spondyloarthritis are of interest since, according to Greek data, the long-term retention (5–10 years) of the first TNFi administered to patients with spondyloarthritis is 60% and 49%, respectively.

Dr Kitas analysed recent clinical findings of baricitinib therapy (JAK1/JAK2 Inhibitor), referring to RA-BEYOND, RA-BEAM, RA-BUILD, RA-BEACON). Interestingly, baricitinib is superior to adalimumab (44% vs 35 % decrease of DAS28-CRP at 12 weeks) in patients with adequate response to methotrexate, with overall serious infection incidence being quite low (2,9/100 PYE), including herpes zoster (HZ) (3,2/100 PYE). The role of other similar intracellular signalling pathway inhibitors, such as tofacitinib, and in particularly an overview of phase 3/4 studies, was presented by Dr Daoussis. The safety and efficacy were analysed in DMARD-IR (Inadequate Response), MTX-IR or TNFi-IR RA-patients, showing a sustained improvement in signs and symptoms up to 96 months (based on two long-term extension studies). Remarkably, the incidence rate for adverse events of special interest are comparable to bDMARDs, except for herpes zoster (3.9/100 pt-years). In addition, laboratory monitoring recommendations were provided (complete blood counts, lipids, liver function tests). Reassuring data about tofacitinib safety from an analysis of data from global clinical trials (extension up to 8.5 years) and real-world observational data (registries), without new safety risk, was also provided by Dr Ioannis Papadopoulos. However, emerging questions were raised by Dr Katerina Hatzidionisiou, concerning the exact role of these small molecules in RA treatment in a “post-biologics” era (real-life effectiveness and safety, JAK switching, place in the treatment algorithm, and biomarkers of treatment response).

Insights about Giant Cell Arteritis (GCA) pathogenesis (IL-6-IL17 or IL12-INF-γ cytokine cluster, Notch-Notch ligand), clinical phenotypes (large vessel, cranial type), diagnostic procedures (biopsy, US, MRI, PET/CT), treatment recommendations (BSR, EULAR), long-standing glucocorticoid complications (up to 80% of GCA patients), disease relapses (∼50% at the first year) and novel treatment approaches such as tocilizumab (GiACTA trial, with sustained remission up to 56% up to week 52), were presented by Dr Pinelopi Konstantopoulou.

Clinical data and real-world experience of biosimilars such as SB4 (etanercept) were presented by Dr Taylor. Along with comparable responses in clinical indexes (DAS28, ACR) radiographic protection and safety from clinical trials data, Dr Taylor also reflected on encouraging results from observational data, mainly from DANBIO or German Registry, about switchers to SB4 and better retention than the originator agent.

The usefulness and necessity of different Disease Activity Scores for monitoring RA (DAS28, SDAI, CDAI, MHAQ) and spondyloarthritis patients (ASDAS, BASDAI) were presented by Dr Prodromos Sidiropoulos, who concluded that these are important tools in daily clinical practice for quantifying inflammation and optimizing prognosis.

Undoubtedly, the 26^th^ Panhellenic Rheumatology Congress was a great success. Its programme included 66 outstanding lectures and 77 impressive poster presentations, addressing a wide spectrum of issues in rheumatology. All participants look forward to attending the next Panhellenic Rheumatology Congress, a major event for researchers and practitioners encountering various rheumatic and allied health conditions.

